# Reframing Perceptions in Restorative Dentistry: Evidence-Based Dentistry and Clinical Decision-Making

**DOI:** 10.1155/2021/4871385

**Published:** 2021-12-31

**Authors:** Ayah A Al-Asmar, Ahmad S Al-Hiyasat, Motasum Abu-Awwad, Hakam N Mousa, Nesreen A Salim, Waed Almadani, Furat Rihan, Faleh A Sawair, Nigel B Pitts

**Affiliations:** ^1^Department of Conservative Dentistry, School of Dentistry, University of Jordan, Amman, Jordan; ^2^Department of Conservative Dentistry, School of Dentistry, Jordan University of Science and Technology, Irbid, Jordan; ^3^Department of Prosthetic Dentistry, School of Dentistry, University of Jordan, Amman, Jordan; ^4^Department of Oral Pathology, Faculty of Dentistry, University of Jordan, Amman, Jordan; ^5^Dentist, Private Sector, University of Jordan, Amman, Jordan; ^6^Dental Innovation and Impact, Faculty of Dentistry, Oral & Craniofacial Sciences King's College, London, UK

## Abstract

**Objectives:**

The worldwide interest of both dentists and patients in esthetic dentistry has affected decision-making in dental practice. The aim of this study was to investigate contemporary dental practice in restorative dentistry and the relationship between evidence-based dentistry in caries research and decision-making in clinical practice in restorative dentistry.

**Methods:**

The study was conducted through a structured questionnaire distributed randomly at the Jordanian Dental Association registered dentists in Jordan. The questionnaire aimed to clarify the degree of knowledge and practice of evidence-based dentistry in caries research the dentists hold regarding clinical decision-making in restorative dentistry.

**Results:**

The majority of the surveyed dentists (77%) treat teeth with irreversible pulpitis with root canal treatment rather than vital pulp therapy. 13.8% routinely insert a post and 23% routinely crown the tooth after root canal treatment regardless of the remaining tooth structure. Badly damaged teeth are treated with full crowns in 72% of the cases. Regarding Hollywood smile or smile makeover, the majority of dentists choose conservative approaches, and implants were the first choice to replace missing teeth for 93.8% of the surveyed dentists.

**Conclusion:**

A higher degree of implementation of evidence-based dentistry in clinical decision-making was found in Prosthetic Dentistry than in Endodontics. Yet, the gap between evidence-based data and clinical practice needs bridging. More emphasis on communicating these data to educators to integrate them into the dental curriculum is a must.

## 1. Introduction

Worldwide, there has been an intensive interest in esthetic profile in general, and in esthetic dentistry in particular. Patients seeking “Hollywood smile” and “smile makeover” are dominating those who are concerned with their oral health. On the other side, dentists seeking fulfilling patients' demands are predominating those who are seeking fulfilling patients' needs.

Thus, the practice of dentistry has become increasingly commercialized and commodified due to conflicts between the commercial and professional obligations that dental practitioners face every day [1]. Moreover, patients' perception of facial beauty and esthetic dental appearance (Hollywood smile makeover) has become inspired by the beauty displayed by movie actors and social media effects rather than health or scientific reasoning [2]. We discussed previously the ethical and the scientific philosophies that lie behind the key concept of Minimal Invasive/Intervention Dentistry (MID), and we emphasized the need for updating dentists' practice dictionary with a new set of terms and norms [3]. The main goal of MID is to increase the life of the teeth, which was restored with less intervention conveying the concept “prevention of extension” rather than “extension for prevention” [4]. The scientific advances in the knowledge of the caries process combined with developments both technological and technique related, impose that the sole rational pertinent therapeutic model is one that is based on prevention and treatment using the least invasive approaches and MID [5]. Although this concept was evolved over decades, the answer for the question: ‘Are we ready to move from operative to nonoperative/preventive treatment of dental caries in clinical practice?' [6] remained over decades ‘No' due to the disconnection between accepted international evidence-based dentistry (EBD) on how best to prevent and manage caries and the care delivered in dental practice [7]. This miscommunication and the big gulf between research findings and clinical practice led to the wasting of evidence-based practice in patient dental care.

Today and after the emergence of the COVID-19 pandemic, there is a further essential need for MID implementation and a great emphasis should be placed on the importance of incorporating the Minimal Invasive Oral Care (MIOC) term in primary dental care in daily clinical practice and across all dental disciplines [8]. Shifting the paradigm to

1. )Minimizing Aerosol Generating Procedures (AGP) and aggressive operative intervention across all dental disciplines
2. Maximizing the utility of MIOC and preventive oral health philosophies and approaches, which should become a funded aspect of primary care delivery globally
3. Implementing new norms routinely in daily dental practice (online personalized preventive oral health advice, teledentistry, protective personal equipment (PPE), and airborne precautions) helping to evolve the relationship between dental practices and their patients and enhance primary oral healthcare services
4. Increase the awareness in populations of their role in valuing and taking responsibility for their personal healthcare future
5. Develop and coordinate clinical global MIOC guidelines or framework to reshape the clinical strategies and protocols across the dental disciplines

The aim of this study was to investigate contemporary dental practice in restorative dentistry and the relationship between EBD in caries research and decision-making in clinical practice in restorative dentistry.

Null hypothesis: there is no correlation between EBD in caries research and decision-making in clinical practice in restorative dentistry.

### 1.1. Endodontically Treated Teeth

With advanced knowledge and research in pulp biology and restorative treatment, many of the concepts that we were practicing have been altered to be more conservative clinical approaches. This includes the condition of the pulp statues with symptoms and how to manage it, as well as the way to restore the endodontically treated teeth.

It is well known that dental pulp is not a doomed organ. It has the ability to initiate several defense mechanisms to protect itself from bacteria to a certain extent [9]. The concept that regarded the pulp as irreversibly inflamed whenever a carious exposure occurs in mature permanent teeth has been based on clinical outcomes of direct pulp capping with calcium hydroxide [10].

The better understanding of pulp biology and the regenerative process, and the healing potential of the inflamed pulp has encouraged the moving towered vital pulp therapy (VPT) approach in selected cases [11, 12]. Indeed, studies have shown evidence by a histological test that the inflammation is confined to a limited area of the pulp within the exposure site, and it is not uncommon to find normal histological structure in the coronal pulp away from caries as well as in the radicular pulp [13, 14]. Therefore, in recent years, the VPT has been increasingly considered as a minimally invasive approach for the management of teeth with inflamed pulps compared to the conventional approach of root canal treatment (RCT) [15–17]. However, some clinicians still prefer to go for conventional RCT, as a more warranty approach, and to avoid the risk of unsuccessful VPT, although the RCT is more aggressive treatment and costs more for the patient.

The other dilemma for endodontically treated teeth is how to restore these teeth after the root canal treatment since most of the time, these teeth are with large caries and loss of coronal tooth structure. Indeed, the best choice of the treatment protocol to restore the endodontically treated teeth is still unclear, and there are many factors and choices to be considered, including the necessity for the post, the type of coronal restoration, the amount of remaining coronal tooth structure, and even the type of luting agent used [18]. Although the postinsertion helps to enhance the retention of the coronal restoration and the crown insertion, which should protect the remaining tooth structure, a recent laboratory study reported that the nonrestorable fractures that may occur in endodontically treated teeth only occurred in teeth restored with posts. The authors suggested that composite buildups without posts may be an option for restoring endodontically treated incisors with 2 mm ferrule height [19]. Therefore, the option for restoration of endodontically treated teeth should be taken with care for each case as such. It was reported that restorative preferences related to the use of posts have changed over time, and it seems to be influenced by the experience of the clinician and the postgraduate training that the dentists have received [18].

### 1.2. Cuspal Coverage for Weakened Teeth

With the advances in adhesive dentistry, multiple restorative treatment options are available to restore endodontically treated teeth (ETT) [20]. Variations in decision-making between clinicians regarding the management of posterior ETT are still evident to this day, and there is still no consensus regarding when cuspal coverage might be needed compared with other more conservative treatment options [21].

ETT are at higher risk of fracture than intact teeth [22]. This is attributed mainly to the loss of tooth structure which is directly associated with tooth strength and resistance to fracture [23]. The reduction in the protective feedback mechanism in these teeth could also put such teeth at higher risk of receiving increased occlusal loads, which possibly makes them more prone to fractures [24].

There is still no consensus in the literature regarding the best choice of restoration for ETT. Evidence from the literature had reported full cuspal coverage to provide better longevity for these teeth than intracoronal restorations [25–27]. However, other reports in the literature had advocated more conservative treatment options. In one prospective 3-year clinical study, no significant clinical difference was found between full cuspal coverage and intracoronal composite restorations when restoring endodontically treated premolars with MO/DO cavities that had preserved cuspal structure [28]. Other studies reported 78% survival rate at 5 years for endodontically treated molars with an occlusal cavity restored with intracoronal restorations and 80% survival rate at 3-years for endodontically treated premolars with a MO/DO cavity and axial wall thickness of >2.5 mm [29, 30].

The restoration of ETT with cuspal coverage when other more conservative treatment options would suffice could be considered an overtreatment. Factors that influence the need for cuspal coverage include the amount and distribution of tooth structure remaining [31, 32], the type and amount of load applied on the tooth during function [33], the parafunctional habits of the patient [34], the esthetic value of the tooth, and the knowledge and experience of the dentist [35]. Therefore, each clinical situation should be evaluated separately, and the final decision should be customized for each case.

### 1.3. Minimal Invasive Alternatives to Hollywood Smile

As practitioners in the field of Restorative Dentistry to enhance and beautify smiles, understanding the difference between Esthetic (Aesthetic) and Cosmetic Dentistry will be the first step when treatment planning dental cases. This will give us the tools to confine treatment options within the needs and wellbeing of our patients [36]. Cosmetic and esthetic dentistry is different in definition, concept, and execution; cosmetic dentistry is commonly selected as an interim procedure that does not necessarily function ideally and does not always emulate the pristine state of natural dentition, while esthetic dentistry requires less accommodation, incorporates acceptable biologic technology for long-term survival, functions suitably, and mimics the pristine state of the natural dentition [37]. There are a variety of dental options to reach an esthetic outcome, ranging from “no treatment” option as the most conservative solution to more aggressive options such as full coverage restorations, extractions, or implants. Many cases can be treated with the MID approach. Teamwork is the key factor in planning such cases. Traditional orthodontic treatment to align teeth then using the option of whitening procedure could be enough to reach functional and esthetic results without changing the integrity of the enamel surface: the development of tooth whitening and advanced restorative and prosthetic materials and techniques, supported by the pioneering discovery of dental adhesion; the significant progress in orthodontics and periodontal and oral and maxillofacial surgery; and, most recently, the implementation of digital technologies in the 3-dimensional planning and realization of truly natural, individual, and esthetic smiles [38].

Composite resins offer a solution when restoring teeth in the esthetic zone, and their benefits include strength, esthetics, and a lower cost than glass ceramic restorations. When used as an additive type of restoration, the greatest advantage is that they are almost completely reversible. If the patient is not satisfied with the restoration, they can be removed with minimal damage to the natural structure [39].

Our goal as restorative dentists should focus on keeping tooth integrity with minimal alterations or modifications. Patients seeking more visual changes to their smiles can be treated with ceramic or composite veneers with traditional, minimal, and selective or no preparation designs. Using wax-ups and mockups will help dentists, lab technicians, and patients to be on the same page, reaching outcomes that are esthetically predictable and realistic. This workflow method could be fabricated traditionally by the lab technician or digitally using intraoral scanners, smile design software, and 3D printing. Modifying the use of mockups as a preparation guide for dentists or as a surgical guide for periodontists will ensure more conservative soft and hard tissue manipulation with minimally invasive approach.

## 2. Materials and Methods

According to the ethics policy of the University of Jordan, the ethical approval form and permission to collect the needed data were signed and approved by the Faculty of Dentistry Research and Ethics Committee (FDREC) and the Academic Research Committee (ARC) at the University of Jordan.

The Jordanian Dental Association (JDA) database registered (7012) dentists as working dentists in Jordan, in which there are (5406) general dentists and (405) specialists in restorative dentistry. The study was conducted through a structured questionnaire (See Supplementary file) that was generated using the SurveyMonkey website to be distributed randomly via web survey to these dentists in different dental sectors (private sectors/Ministry of Health/Universities/Royal Medical Services). The inclusion criteria were as follows: at least two years' experience as working practitioners, general practitioners together with specialists in restorative dentistry (operative dentistry, endodontics, and fixed prosthodontics). The sample size was composed of 5811 dentists whom we could reach via Internet (e-mail, messenger, what's app). 4000 responses were collected from the web survey, response rate (68.83%).

The questionnaire was validated for validity and reliability by distributing it to 10 dentists (restorative dentistry specialists) out of the sample size. The questionnaire was then modified and adjusted according to these 10 dentists' feedback. The questionnaire consisted of sociodemographic and professional characteristics such as year of graduation, name of country/university of bachelor degree graduation, expertise years, and the specialty if one exists. The dentists were asked regarding the following topics:

1. )Whether they treat teeth diagnosed with irreversible pulpitis with vital pulp therapy if applicable, or root canal treatment as it is the best predictable successful treatment
2. Whether they routinely insert a post in endodontically treated teeth routinely for protection, regardless of the remaining tooth structure or other related factors
3. Whether they routinely crown any tooth after root canal treatment for protection, despite the remaining tooth structure
4. Whether they treat badly damaged teeth with direct composite or amalgam restorations, intracoronal restorations (inlays/onlays), or with full cuspal coverage (full crowns)
5. Whether they treat anterior teeth with veneers as the first choice of treatment, or they offer the patient other choices such as bleaching, orthodontic treatment, and dental composite veneering, or they refuse treatment if unnecessary
6. Whether they choose the color (shade) of the anterior veneers they do for their patients according to the clinical condition of the patient, or they obey the patients' preferences
7. Whether they convince the patient to replace a missing tooth with an implant as the first choice of treatment or with a bridge

The collected information and responses were coded and statistical analysis was performed using the software SPSS Statistics for Windows, Version 16.0 (SPSS Inc., Chicago, IL, USA). All data were tested for normality using the Shapiro-Wilk test. Descriptive statistics were generated and the chi-square test was used to examine associations between the different variables. The significance level was set at *P* < 0.05.

## 3. Results

The overall response rate was 68.83% (4000 of 5811 potential participants). The demographic characteristics of the study population are presented in Table 1.

As shown in Table 2, the majority (77%) treat teeth with irreversible pulpitis with root canal treatment but 23% treat such teeth with vital pulp therapy if applicable. Vital pulp therapy was applied more frequently by dentists graduated from West Europe, those with more than 20 years of experience, and endodontists.

When dealing with root canal treated teeth, 13.8% routinely insert a post regardless of the remaining tooth structure. This was practiced more frequently by male dentists, those with more than 20 years of experience, and particularly those who graduated from East Europe and less frequently by endodontists and those working at universities. Additionally, 23% routinely crown any tooth after root canal treatment for protection regardless of the remaining tooth structure. Similarly, this procedure was conducted more frequently by male dentists, those who graduated from East Europe, general dental practitioners, and those working at the Ministry of Health.

Of the surveyed dentists, 72% treat badly damaged teeth with full crowns, while 14.5% treat with amalgam or composite restorations, and 13.5% with inlays or onlays. Amalgam/composite restorations were used more frequently by females, those with more than 20 years of experience, and those working at the Ministry of Health, and less frequently by endodontists. Graduates from West Europe/USA and prosthodontists used inlays/onlays more frequently.

When the patient asks for a Hollywood smile or a smile makeover, 6.5% offer the porcelain/ceramic veneers, 43.5% offer bleaching, orthodontic treatment, or composite veneers, while 50% offer no treatment if unnecessary. Males, those with more than 20 years of experience, and dentists working at Royal Medical Services offer porcelain/ceramic veneers more frequently.

When doing anterior veneers, the majority (79.5%) discuss the color (shade) with patients and convince them for the best, despite the patients' preferences. On the other hand, 9.5% choose the color according to the clinical condition of the patient, and 11% let the patients choose the color. Significantly higher percentage of dentists who graduated from East Europe choose the color according to the clinical conditions of the patients.

Without significant effects of the sociodemographic variables, implants were the first choice to replace missing teeth of 93.8% of surveyed dentists, while 6.2% preferred a bridge as their first choice.

## 4. Discussion

With the current changeover in all dimensions of dental caries in the twenty-first century, productive and desirable changes are mandatory in the daily practice of clinical decisions [40]. In the current study, the null hypothesis was rejected in most of its facets. Esthetic and replacement treatments followed conservative approaches, while endodontic and badly damaged teeth treatments adopted aggressive interventions routinely.

VPT is a conservative treatment option that aims to preserve the vitality and function of the remaining pulp tissue in vital teeth through various techniques, including indirect or direct pulp treatment, partial or complete pulpotomy [41]. Although evidence-based endodontics considers VPT a successful, less expensive, and simpler alternative in modern dentistry, the majority of dentists prefer to treat teeth with irreversible pulpitis with conventional root canal treatment [42, 43]. Undergraduate curriculum, postgraduate studies, and years of experience are the variables associated with the treatment option which was adopted in the current study. Perhaps the associated success-determining factors such as judgment criteria and pulp status at the time of treatment, vascularization of the pulp and its healing potential, age of the pulp, a nominated best therapeutic technique, and well-established comprehensible diagnostic indications make it harder for the clinician to adopt such a strategy [42–45]. Endodontics hold a level of knowledge about indications regarding various pulpal treatments finer than general practitioners [46]. Therefore, competent dentists (endodontists and experienced dentists) have had more courage to select the appropriate cases to perform more conservative yet untraditional treatments via VPT, rather than routinely treating irreversible pulpitis with conventional RCT. On the other hand, a recent multicentered study demonstrated different results in which general practitioners performed more VPT than RCT in comparison with endodontics [47]. Unfortunately, in spite of outlining the microbiological etiology of pulpal diseases and testing the capability of pulpal healing, controversies encountered in the endodontic field is the lack of understanding of the nature of the disease process and healing pathways [45, 48]. The schism between clinicians and scientists is propagated by a tendency of each group to confer with themselves rather than with each other, thus accumulated biological knowledge did not find clinical application in the endodontic arena [45, 49].

The restoration of ETT may represent another challenge for endodontics and restorative dentists as there is no consensus on the best treatment/restoration after RCT [50]. The remaining coronal tooth structure and the functional requirements are critical factors in clinical decision-making regarding the restoration of such teeth [51]. The best method of treatment aims to achieve greater longevity of the brittle tooth and to increase its fracture resistance [50, 52]. 13.8% of our dentists routinely insert a post and 23% crown an ETT regardless of the remaining tooth structure. Endodontists and dentists working at universities practiced posting an ETT less frequently. General dental practitioners and those working at the Ministry of Health practiced crowning an ETT more frequently. Of the surveyed dentists, 72% treat badly damaged teeth with full crowns, while prosthodontics used inlays/onlays more frequently. These results, although indicating that the scientific background of the clinician affects his or her clinical decision in practice contradict the results of Turp and his colleagues which found a preference for endodontics to post and crown ETT [51]. The scientific evidence-based dentistry/endodontics regarding the best method of treating ETT is debatable and not clear due to lack of randomized clinical studies that can be compared, the continuous emergence of new adhesive materials and bonding procedures, various treatment strategies adopted by clinicians according to different clinical experience, and inconsistent, conflicting results with various materials and different experimental designs [50, 52–54]. Nevertheless, it is clearly stated that the remaining tooth structure and the permanent coronal seal play a vital role in determining the long-term success of the final restoration [50, 54–56], the position of the tooth in the arch affects the necessity of posting the tooth or not [55, 57], and such tooth can benefit from the advances of adhesive materials for cementing the posts and the cuspal coverage [52–54, 56].

Esthetic dentistry has transformed the patients' perspectives of dentistry dramatically. There is a growing demand for esthetic dental treatments seeking the ideal smile being advertised through media [58]. However, many studies showed that dentists are far more critical in their esthetic perceptions compared to patients in general [58–60]. The authors of this article believe that dentists should participate actively in the treatment plan of their patients even if the end result was offering the patient no esthetic treatment if not needed. Half of the surveyed dentists in the current study offer no treatment if unnecessary to patients seeking a smile makeover, and the majority of them discuss the shade with their patients despite the patients' preferences. Although it is known that lay people prefer smiles with lighter teeth shade as teeth color is a highly significant factor in the perception of smile attractiveness [61], dentists should not always give the patients what they want serving the market if such treatment is against their scientific background and values [62].

Our dentists chose a more conservative approach regarding replacing missing teeth with implants vs. bridges. However, this result is highly expected not only due to the advantage of allowing preservation of the integrity of other sound teeth adjacent to the edentulous area but also because the use of osseointegrated implants has become a well-established and predictable treatment providing a highly esthetic, functional, and long-term result [63].

The limitations of the current study in the aspect of gathering data are due to the COVID-19 pandemic, in which collecting data solely depended on the electronic survey. The questionnaire was designed to be as concise as practical to ensure a high rate of responses. More sophisticated investigations that can lead to a deeper insight into the study can be carried on later. In terms of statistical analysis, descriptive statistics were used as this research was the first time to be done in the cariology era in Jordan.

## 5. Conclusion

Regarding the restorative procedures proposed in the current study, clinical decision-making in dental practice is highly influenced by the clinician's own knowledge background and clinical experience. The lack of generalized or standardized treatment strategies in the literature with the many inconsistent results adds difficulty to utilizing evidence-based dentistry when adopting a treatment strategy regarding these restorative procedures.

## Figures and Tables

**Table 1:** Sociodemographic characteristics of the studied sample.

Variable		Number (%)
Gender	Male	1720 (40)
	Female	2280 (57)
Country of last degree	Jordan	2430 (60.8)
	Other Arab/Asian countries	830 (20.80
	West Europe/USA	530 (13.2)
	East Europe	210 (5.2)
Experience (years)	<5 years	1050 (26.2)
	5–10 years	1070 (26.8)
	11–20 years	1050 (26.2)
	>20 years	830 (20.8)
Training status	General practitioner	2730 (68.2)
	Conservative dentistry	320 (8)
	Endodontics	510 (12.8)
	Prosthodontics	440 (11)
Working place	Private clinic/center	3040 (76)
	University	390 (9.8)
	Ministry of health	370 (9.2)
	Royal medical services	200 (5)

**Table 2:** Routine dental treatments and its association with sociodemographic variables.

Condition	Treatment options	Total %	Gender		Country of last degree				Experience (years)				Training status				Working place			
			Male	Female	Jordan	Other Arab/Asian countries	West Europe/USA	East Europe	<5	5–10	11–20	>20	GDP	Conservative dentistry	Endodontics	Prosthodontics	Private clinic/center	University	MOH	RMS
		%																		
I treat teeth with irreversible pulpitis with	Vital pulp therapy (if applicable	23	21.5	24.1	22.6	20.5	35.8	4.8	13.3	21.5	21.9	38.6	19.4	25	41.2	22.7	21.7	38.5	18.9	20
	RCT (as best predictable successful treatment)	77	78.5	75.9	77.4	79.5	64.2	95.2	86.7	78.5	78.1	61.4	80.6	75	58.8	77.3	78.3	61.5	81.1	80
	*P* value		0.539		**0.027**				**0.001**				**0.009**				0.112			
I routinely insert a post for root canal treated teeth regardless of the remaining tooth structure	Yes	13.8	22.1	7.5	5.3	28.9	9.4	61.9	5.7	15.9	11.4	24.1	17.6	6.2	2	9.1	15.8	0	16.2	5
	No	86.2	73.9	92.5	94.7	71.1	90.6	38.1	94.3	84.1	88.6	75.9	82.4	93.8	98	90.9	84.2	100	85.8	95
	*P* value		**<0.001**		**<0.001**				**0.003**				**0.009**				**0.033**			
I routinely crown any tooth after RCT for protection regardless of the remaining tooth structure	Yes	23	29.7	18	15.6	41	18.9	47.6	14.3	28	21.9	28.9	27.8	9.4	13.7	13.6	24.3	5.1	35.1	15
	No	77	70.3	82	84.4	59	81.1	52.4	85.7	72	78.1	71.1	72.2	90.6	86.3	86.4	75.7	94.9	64.9	85
	*P* value		**0.006**		**<0.001**				0.052				**0.009**				**0.011**			
I treat badly damaged teeth with	Direct restoration with composite or amalgam	14.5	9.9	18	15.2	15.7	9.4	14.3	19	8.4	9.5	22.9	17.6	18.8	2	6.8	14.1	7.7	24.3	15
	Intracoronal restorations (inlays/onlays)	13.5	19.8	8.8	7.8	16.9	35.8	9.5	5.7	15.9	22.9	8.4	8.4	31.2	15.7	29.5	11.5	35.9	5.4	15
	Full cuspal coverage (full crowns)	72	70.3	73.2	77	67.5	54.7	76.2	75.2	75.7	67.6	68.7	74	50	82.4	63.6	74.3	56.4	70.3	70
	*P* value		**0.001**		**<0.001**				**<0.001**				**<0.001**				**0.001**			
When the patient asks for a Hollywood smile or smile makeover	I offer porcelain/ceramic veneers on the upper or/and lower anterior as it is the first choice of treatment	6.5	11	3.1	2.9	13.3	5.7	23.8	6.7	10.3	4.8	3.6	7	9.4	0	9.1	7.2	0	2.7	15
	I offer him/her other choices such as bleaching, orthodontic treatment, and/or dental composite veneering	43.5	40.7	45.6	48.6	30.1	45.3	33.3	48.6	48.6	40	34.9	44	37.5	39.2	50	47.7	38.5	24.3	25
	I offer no treatment at all if unnecessary	50	48.3	51.3	48.6	56.6	49.1	42.9	44.8	41.1	55.2	61.4	49.1	53.1	60.8	40.9	45.1	61.5	73	60
	*P* value		**0.006**		**<0.001**				0.065				0.302				**0.004**			
When I do anterior veneers for patients	I choose the color (shade) according to the clinical condition of my patient	9.5	12.8	7	7.4	6	15.1	33.3	11.4	7.5	7.6	12	9.2	6.2	11.8	11.4	9.2	17.9	5.4	5
	I let my patients choose the color (shade)	11	12.2	10.1	12.8	8.4	5.7	14.3	12.4	11.2	12.4	7.2	10.6	9.4	17.6	6.8	11.2	7.7	10.8	15
	I discuss the color (shade) with my patients and I convince them for the best, despite his/her preference	79.5	75	82.9	79.8	85.5	79.2	52.4	76.2	81.3	80	80.7	80.2	84.4	70.6	81.8	79.6	74.4	83.8	80
	*P* value		0.100		**0.001**				0.747				0.631				0.551			
I convince the patient to restore the missing tooth with	An implant as first choice of treatment	93.8	95.3	92.5	93.4	96.4	94.3	85.7	94.3	95.3	96.2	88	92.7	90.6	98	97.7	93.8	94.9	91.9	95
	A bridge	6.2	4.7	7.5	6.6	3.6	5.7	14.3	5.7	4.7	3.8	12	7.3	9.4	2	2.3	6.2	5.1	8.1	5
	*P* value		0.251		0.337				0.096				0.227				0.949			

The significance level was set at *P* < 0.05. The bold values are *P* values that are less than 0.05 for each set of data for each question.

## Data Availability

All data generated and analyzed in this article are included within the article.

## Supplementary Materials

Supplementary Materials The questionnaire that was used in this study to investigate if a relation exists between evidence-based dentistry and clinical decision-making in restorative dentistry (supplementary file 1).
