# Supplementary material for: Reframing Perceptions in Restorative Dentistry: Evidence-Based Dentistry and Clinical Decision-Making
Source: Int J Dent. 2021 Dec 31;2021:4871385. doi: 10.1155/2021/4871385 (PMC8741395; doi:10.1155/2021/4871385)
Supplement: Supplementary Materials — The questionnaire that was used in this study to investigate if a relation exists between evidence-based dentistry and clinical decision-making in restorative dentistry (supplementary file 1). [file 4871385.f1.docx]

**Evidence-Based Dentistry and Clinical Decision Making**

This survey is part of a research to investigate if a relation exists between evidence-based dentistry and clinical decision making in restorative dentistry.

This questionnaire will only take 2-3 minutes of your time to complete and will ask you just 18 questions. Information provided is confidential and the responses are kept as anonymous.

**1- Gender**

Male

Female

**2- Country of last degree/certificate obtained**

Drop down list

**3- Years of experience in dental practice**

< 5years

5-10years

11-20years

>20years

**4- Training status**

General practitioner

Specialist (Conservative Dentistry)

Specialist (Endodontics)

Specialist (Prosthodontics)

**5- Work place**

Private clinic/centre

University

Ministry of Health

Royal Medical Services

**6- I treat teeth diagnosed with irreversible pulpitis with:**

**أعالج الأسنان/الأضراس المشخصة بالتهاب العصب السني النهائي:**

1- Vital pulp therapy (if applicable) بواسطة علاج العصب اللبي الحيوي إن أمكن

2- Root canal treatment (as best predictable successful treatment)

بواسطة بتر العصب الكلي حيث أنه أنجح علاج ونتائجه قابلة للتنبؤ

**7- I routinely insert a post for endodontically-treated tooth for protection, regardless the remaining tooth structure or other related factors.**

عادة ما أستخدم الوتد للأسنان التي عولجت ببتر العصب الكلي لحمايتها، بغض النظر عن كمية المادة السنية المتبقية أو العوامل المرتبطة الأخرى.

1- Yes

2- No

**8- I routinely crown any tooth after root canal treatment for protection, despite the remaining tooth structure.**

عادة ما أقوم بوضع تاج على الأسنان التي عولجت ببتر العصب الكلي لحمايتها، بغض النظر عن كمية المادة السنية المتبقية.

1- Yes

2- No

**9- When the patient asks for Hollywood smile or smile makeover:**

**عندما يطلب المريض ابتسامة هوليوود أو ابتسامة المشاهير:**

1- I offer him/her porcelain/ceramic veneers on the upper or/and lower anterior teeth as it is the first choice of treatment in such conditions

أعرض عليه/عليها وجوه خزفية للأسنان الأمامية العلوية و/أو السفلية كأول خيارفي هكذا حالات

2- I offer him/her other choices such as bleaching, orthodontic treatment, and/or

dental composite veneering

أعرض عليه/عليها خيارات أخرى كتبييض الأسنان، تقويم الأسنان، وجوه من الحشوات البيضاء التجميلية

3- I offer no treatment at all if unnecessary

أعرض عليه/عليها عدم المعالجة إن اعتقدت أنها غير ضرورية

**10- When I do anterior veneer for my patients:**

**عندما أقوم بعمل وجوه خزفية للأسنان الأمامية لمرضاي:**

1- I choose the color (shade) according to the clinical condition of my patient

أنا من يقوم باختيار اللون المناسب وفقا لحالة المريض

2- I let my patients choose the color (shade)

أدع مرضاي يختارون اللون الذي يفضلون

3- I discuss the color (shade) with my patients and I convince them for the best, despite his/her preference

أناقش مع مرضاي موضوع اللون حتى أقنعه/أقنعها بالأنسب لحالته/لحالتها بغض النظر عما يفضلونه

**11- I treat badly damaged teeth with:**

**أعالج الأسنان/الأضراس المتضررة بشكل كبيربواسطة:**

1- Direct restoration with composite or amalgam

حشوات بيضاء تجميلية راتنجية أو حشوات فضة مباشرة

2- Intracoronal restorations (inlays/onlays) حشوات الترصيع المصنعة مخبريا

3- Full cuspal coverage (full crowns) التيجان

**12- I convince the patient to replace a missing tooth with:**

**أقنع المريض بتعويض السن/الضرس المفقود من خلال:**

1- An implant as first choice of treatment زراعة الأسنان كأول خيار علاجي

2- A bridge صناعة جسر ثابت

**Many thanks**
